# 2390. Factors Affecting Parental COVID-19 Vaccine Hesitancy in Cancer Parents with Minor Children

**DOI:** 10.1093/ofid/ofad500.2010

**Published:** 2023-11-27

**Authors:** Kazue Umetsu, Kazuhiro Kosugi, Tomoko Takahashi, Daisuke Fujisawa, Takashi Kawaguchi, Emi Kubo, Yujiro Inoue, Keisuke Ishizuka, Mariko Harada, Tomonori Inoue, Kento Ohtake, Shiori Yamaya, Jun Takehana, Takahiro Matsuo, Keiji Okinaka, Takuhiro Yamaguchi, Tomofumi Miura

**Affiliations:** National Cancer Center Hospital East, Kashiwa, Chiba, Japan; National Cancer Center Hospital East, Kashiwa, Chiba, Japan; General Incorporated Association Cancer Parents, Cyuo-ku, Tokyo, Japan; Keio University School of Medicine, Minato-ku, Tokyo, Japan; Tokyo University of Pharmacy and Life Sciences, Hachioji, Tokyo, Japan; National Cancer Center Hospital East, Kashiwa, Chiba, Japan; National Cancer Center Hospital East, Kashiwa, Chiba, Japan; National Cancer Center Hospital East, Kashiwa, Chiba, Japan; National Cancer Center Hospital East, Kashiwa, Chiba, Japan; National Cancer Center Hospital East, Kashiwa, Chiba, Japan; National Cancer Center Hospital East, Kashiwa, Chiba, Japan; Medilead Inc., Shinjyuku, Tokyo, Japan; Medilead Inc., Shinjyuku, Tokyo, Japan; The University of Texas MD Anderson Cancer Center, Houston, TX; National Cancer Center Hospital East, Kashiwa, Chiba, Japan; Tohoku University Graduate School of Medicine, Sendai, Miyagi, Japan; National Cancer Center Hospital East, Kashiwa, Chiba, Japan

## Abstract

**Background:**

Cancer patients are vulnerable to COVID-19 infection. Parents living with their minor children in general are at an elevated risk of infection. Thus, cancer patients with minor children (“cancer parents”) are at high risk of contracting COVID-19 and experiencing a severe course. Vaccination is the best clinical approach for preventing COVID-19 infection. However, still, they may hesitate to get their children vaccinated, as parents without cancer does. This study aims to explore the factors associated with parental COVID-19 vaccine hesitancy in cancer parents.

**Methods:**

This study was a secondary analysis of an online cross-sectional survey conducted from December 2022 to January 2023. The eligible participants were cancer patients whose children were aged between 5 and 17. They were recruited through an online peer-support group. The participants were asked to self-report sociodemographic characteristics, medical history, previous COVID-19 infection, vaccination status, fear of COVID-19 scale, and the reasons for COVID-19 vaccine hesitancy. We conducted a multivariate logistic regression analysis to explore the factors associated with COVID-19 vaccine hesitancy. The factors were selected based on a literature review, and variables with a p-value < 0.1 in univariate analysis were retained in the final regression model.

**Results:**

Overall, 199 participants were included [84.9% female; mean age 45.8 years]. The participant’s COVID-19 vaccination rate was 90.5 % (n = 180), but 95 participants (47.7%) were hesitant to vaccinate their child. The factors positively associated with vaccine hesitancy were a younger age of child (adjusted odds ratio (aOR) = 0.66 (95% confidence interval (CI): 0.56–0.77); p< 0.001), lower family income (aOR = 0.22 (95% CI: 0.08–0.62); p=0.004), and fewer participant’s vaccination doses (aOR = 0.34 (95% CI: 0.23–0.50); p< 0.001). The main reasons for vaccine hesitancy were concerns about safety and adverse reactions of the vaccines (Figure 1).

**Figure:**
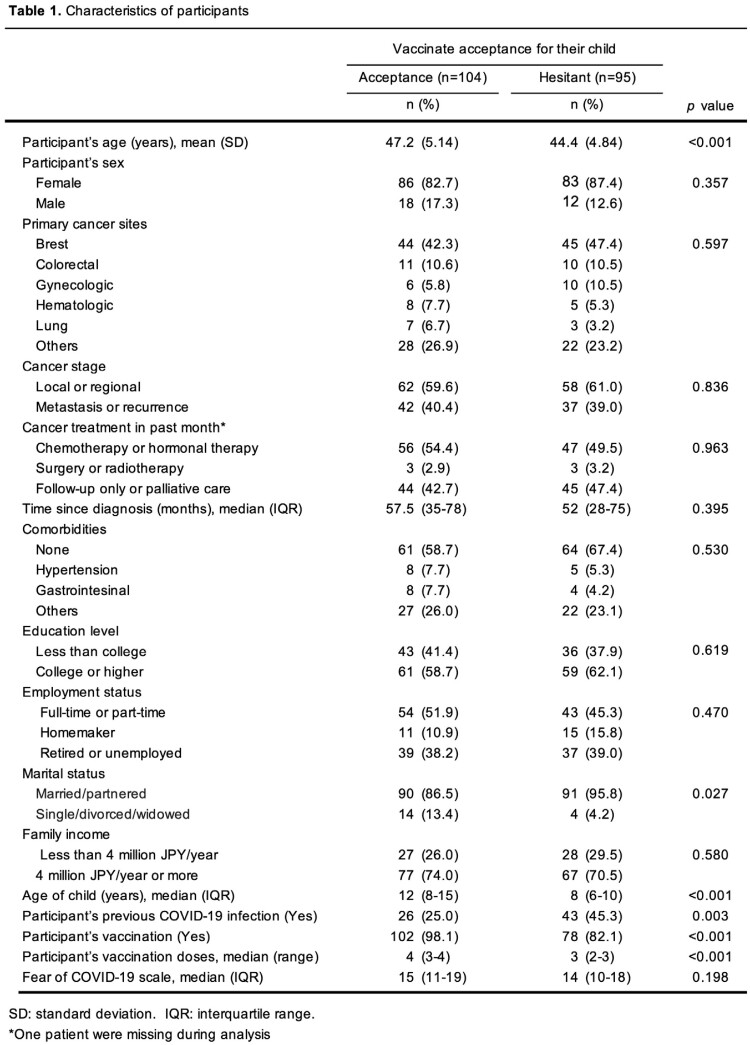

**Figure:**
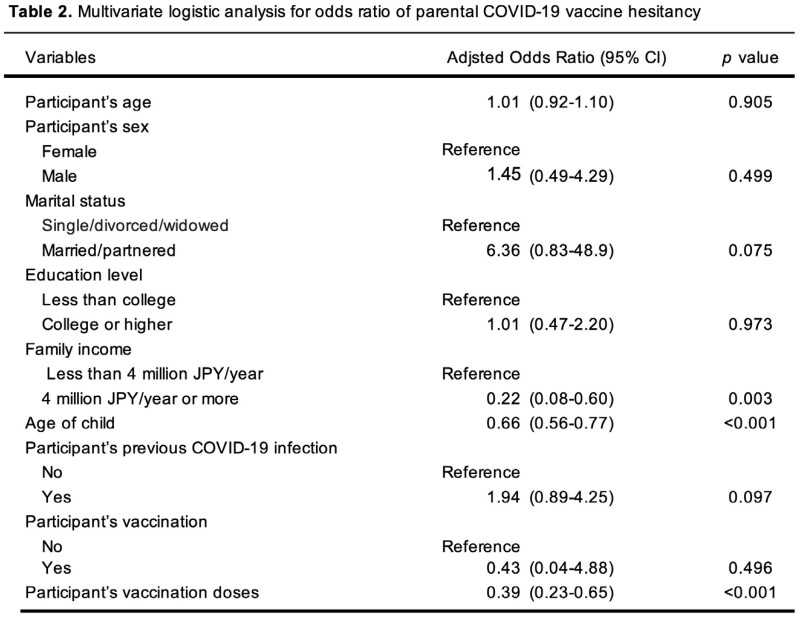

**Figure:**
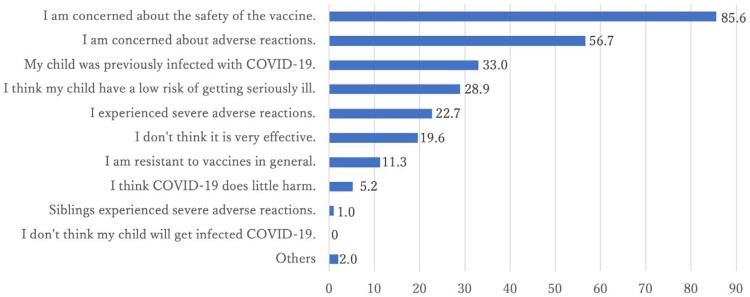

**Conclusion:**

Although most cancer parents had been vaccinated themselves, approximately half of them were hesitant to get their children vaccinated. It is essential to provide cancer parents with detailed information regarding the safety of COVID-19 vaccines to minimize vaccine hesitancy toward their children.

**Disclosures:**

**All Authors**: No reported disclosures

